# Sufficiency and consistency of antenatal care services in Ethiopia: Evidence from Mini Ethiopian demographic and health survey

**DOI:** 10.1371/journal.pone.0319291

**Published:** 2025-06-06

**Authors:** Dessalegn Tamiru, Mohammed Areb, Gemechis Gunja, Million Abera, Desta Asefa, Gurmesa Tura Debelew, Negalign Berhanu, Yibeltal Siraneh, Fira Abamecha, Biru Abdissa Mizana

**Affiliations:** 1 Faculty of Public Health, Institute of Health, Jimma University, Jimma, Ethiopia; 2 College of Medicine and Health Science, Haramaya University, Harar, Ethiopia; 3 Faculty of Health Sciences, Institute of Health, Jimma University, Jimma, Ethiopia; 4 Jimma University Medical Center, Jimma University, Jimma, Ethiopia; 5 Faculty of Medicine and Life Sciences, Hasselt University, Hasselt, Belgium; MeU: Mattu University, ETHIOPIA

## Abstract

**Background:**

Antenatal care plays a critical role in promoting the health of mothers and infants. In Sub-Saharan Africa, about 76% of women accessing at least one visit. However, rural areas in Ethiopia face more significant challenges, including traditional beliefs, geographical distance, and the need for more awareness. Despite advancements, ensuring quality ANC for all women necessitates targeted interventions. There are needs of evidence regarding adequacy and consistency ANC services in different regions of Ethiopia. Therefore, study aimed to evaluate and compare the adequacy and consistency of ANC services across various regions in Ethiopia.

**Methods:**

Data for the 2019 Mini Ethiopia Demographic and Health Survey (Mini EDHS) were collected using a cross-sectional study design. The survey included 2,918 women aged 15–49 from various regions of Ethiopia. Data analysis was carried out using STATA version 14. Descriptive statistics, such as frequencies and percentages, were used to present the background characteristics of the participants. To assess the significance of variations and associated factors, a logistic regression model was applied. Variables with a p-value of 0.25 or less in the bivariate analysis were included in the multivariable analysis. A significance level of p < 0.05 and a 95% confidence interval were used to determine the significance of the variables.

**Results:**

The study surveyed 2,918 women aged 15–49, with 70.7% receiving antenatal care (ANC) from a skilled provider. Among them, 56.8% had four or more visits, and 43% started care in the first trimester. Overall, 35.8% received all routine ANC components, and 14.7% received adequate ANC services. ANC adequacy varied by region, with the highest in Addis Ababa (39.1%) and lowest in SNNPR (6.2%). About 72% of women received consistent ANC, with Addis Ababa showing the highest consistency (54.2%). Logistic regression analysis found that education status, region, wealth status, and literacy were significant factors influencing ANC adequacy. Regional disparities showed lower ANC adequacy in Oromia, Benishangul, SNNPR, and Gambela compared to Tigray. Wealthier women and those with secondary or higher education were more likely to receive consistent ANC.

**Conclusion:**

The study underscored the significant impact of regional disparities, financial constraints, education levels, and geographic location on the unequal access to and quality of antenatal care (ANC) services in Ethiopia. Promoting ANC utilization, especially among women with lower education and economic disadvantages, was identified as a key priority. The study recommends targeted interventions to address service gaps for less-educated women and highlights the need for focused efforts to reduce regional disparities in both the adequacy and consistency of ANC services.

## Introduction

Antenatal care is crucial for the health of pregnant women and their babies, occurring during pregnancy. It includes health promotion, screening, diagnosis, and disease prevention. Evidence-based ANC practices have the potential to save lives [1]. Regular ANC visits facilitate early detection and management of pregnancy complications, reducing the risk of maternal and infant deaths. ANC also improves access to skilled birth attendants, ensuring critical assistance during childbirth and promoting positive birth experiences. However, in Africa, ANC services encounter various challenges such as limited healthcare access, inadequate resources, and teen pregnancies [2]. Despite these obstacles, there have been improvements, with an average of 76% of pregnant women in Sub-Saharan Africa receiving at least one ANC visit. New initiatives, like the WHO’s recommendation for a minimum of eight contacts throughout pregnancy, are being implemented. Innovative methods such as mobile clinics and community health workers are being explored, requiring collaboration across multiple sectors [3].

While antenatal care (ANC) coverage has improved in Ethiopia, challenges persist, particularly in rural areas where access is limited. Traditional beliefs and practices, as well as early childbearing, contribute to insufficient ANC services [4–7]. Other obstacles, such as distance, lack of skilled providers, and limited awareness, further hinder access to adequate ANC. Targeted interventions are needed to ensure that all women receive sufficient and complete ANC to maintain good health [8,9].

ANC coverage varies by country, and in Africa, many pregnant women do not meet the World Health Organization’s (WHO) recommendation of four or more ANC visits [3]. Numerous reviews summarize the adequacy of ANC in Ethiopia and discuss its consistency, as well as the variables affecting ANC utilization [1,10]. The WHO updated minimum suggested number of ANC visits in 2016 from four to eight contacts, based on new research indicating that increased interactions between pregnant women and healthcare professionals can lead to fewer maternal deaths and improved pregnancy outcomes and neonatal health [1,6,10,11].

Given the lack of clear evidence regarding regional variations in the adequacy and consistency of ANC service, this study aimed to assess the regional disparity and associated factors using data from the Mini Ethiopian demographic and health survey (Mini-EDHS) 2019.

## Methods

### Data source, study setting, and design

The study was conducted in Ethiopia, the largest country in the Horn of Africa and the second most populous country in Africa. The study utilized data from the 2019 Ethiopia Mini Demographic and Health Survey as its primary data source. The 2019 EMDHS was a cross-sectional survey carried out in nine regional states and two city administrations of Ethiopia from March 21 to June 28, 2019. This study was conducted from January 01 to February 29, 2024.

### Study population

The study population consisted of women who had at least one antenatal care visit during their most recent birth within the five years preceding the 2019 Ethiopian Mini-Demographic and Health Survey.

### Sampling technique

Every region was divided into urban and rural areas according to the 2019 EMDHS, resulting in 21 sampling strata. We selected 305 enumeration areas (EAs) using a probability percentage, with 212 rural and 93 urban locations among them. For effective comparison and proportionate allocation, 25 EAs were chosen from each of the eight regions. Additionally, 35 EAs were selected from the three larger regions (Amhara, Oromia, Southern Nations, Nationalities, and Peoples’ Region). The households in the selected EAs were included in the sampling frame list created between January and April 2019, forming the 2019 EMDHS cluster. Using a specified equal chance probability systematic selection procedure, thirty households were selected from each cluster. A total of 2918 study participants met the requirements for inclusion in this study.

### Data collection procedure and techniques

The data collection tool used for the 2019 EMDHS was questionnaires, and the data collectors were experienced in conducting household surveys. The data were gathered through interviews with women aged 15–49 years, who were long-term residents of the selected households. Essential data for this study were extracted from the 2019 EMDHS data, with a focus on variables related to the adequacy and consistency of antenatal care services across all regions.

### Study variables

The study aimed to examine regional differences in the adequacy and consistency of antenatal care services in Ethiopia. The adequacy and consistency of antenatal care services, as determined by elements including the frequency and timing of ANC visits, the supply of necessary services, and compliance with advised protocols, were the outcome variables of interest. Residence, household wealth index, geographic region, and educational attainment, which were divided into four categories: no education, primary education, secondary education, and higher education, were among the exploratory factors. The study sought to discover potential regional differences in Ethiopia and to obtain insights into the factors impacting the provision of ANC services by analysing these variables.

### Operational definition

#### Timing of first antenatal care visit (early initiation of ANC):

It is an indicator assessing the proportion of pregnant women who initiate ANC within the within three months of gestational age [1].

#### Consistency of ANC visit:

Measured if the pregnant mother received services during antenatal care visits such as, identifying pre-existing health conditions, early detection of complications during pregnancy, health promotion and disease prevention, birth preparedness, and complication planning [12,13].

#### Sufficient antenatal care:

Sufficient antenatal care involves the recommended minimum of four timely visits, providing comprehensive services such as screenings, monitoring, and health counseling to ensure the well-being of both mother and baby [1,12,13].

#### Adequacy of antenatal care:

The adequacy of antenatal care is measured using three indicators: early initiation of antenatal care (the first antenatal care visit made during the first trimester or three months), attending enough visits (at least four and above visits), and getting services at least once during the pregnancy care) [12,13].

#### Wealth index:

The wealth index weights were derived using principal component analysis (PCA) based on data collected from household assets, utilities, and other variables from the Ethiopian Demographic and Health Survey. PCA was used to create latent factors that represent wealth, with the first factor serving as the household wealth score. These scores were then divided into five quintiles: lowest, second, middle, fourth, and highest [9].

### Data quality management and analysis

The study was conducted a thorough examination of the data from the 2019 Ethiopian Demographic and Health Survey (EDHS) for errors, completeness, and consistency with the 2019 EDHS report. The analysis was performed using STATA Version 14. Initially, we identified all the explanatory variables that potentially influenced the dependent variable. The data underwent cleaning, merging, and re-coding to derive relevant variables for analysis.

The essential background characteristics of the study participants were presented using frequencies and percentages. Results were communicated through tables, figures, and text. A logistic regression model was employed to comprehend the significance of variation and associated factors. Variables with a p-value of 0.25 in bivariate analysis were included for multivariable analysis. A significant level of p-value 0.05 with a 95% confidence interval was used to determine the importance of the variables. Overall, the study process ensured the accuracy and reliability of the data analysis, providing valuable insights into regional differences in antenatal care services in Ethiopia.

### Ethical consideration

We obtained a letter of ethics approval from the DHS. It allows us to access the EDHS-2019 data set from (https://dhsprogram.com/Data/). We followed the data-sharing policy and guidelines. We kept the data private. We used it only for this study. You can read the entire report on the ethical issue in the EDHS-2019 [8].

## Results

### Socio-demographic characteristics of the participants

The study included women aged 15–49 years who had live birth in the last year (n = 2918). Nine hundred forty-eight (32.5%) of the women who participated in the study were between 25 and 29 years. More than three-fourth (69.5%) of the women were rural by residence. One thousand two hundred and sixty-nine (43.5%) of respondents had no formal education. Similarly, 1167 (40%) had four or more young children. In the household wealth index, 904 (31%) participants were in the richest wealth quantile “Table 1.”

**Table 1:** Socio-demographic variables of women of reproductive age in Ethiopia, 2019E_DHS (n = 2918).

Variables	Frequency	Percent
Age group (years)		
15-19	169	5.79
20-24	611	20.94
25-29	948	32.51
30-34	600	20.56
35-39	392	13.43
40-44	153	5.24
45-49	45	1.54
Region		
Tigray	322	11.03
Afar	230	7.88
Amhara	336	11.51
Oromia	346	11.9
Somali	86	2.95
Benishangul	299	10.25
SNNPR**	326	11.17
Gambela	268	9.18
Harari	247	8.46
Addis Ababa	227	7.78
Dire Dawa	231	7.92
Place of residence		
Urban	889	30.47
Rural	2029	69.53
Religion		
Orthodox	1089	37.3
Protestant	585	20.1
Muslim	1202	41.2
Others*	42	1.45
Educational level		
No education	1269	43.5
Primary	1066	36.53
Secondary	356	12.2
Higher	227	7.77
Marital status		
Never in union	19	0.7
Currently in union/living with a man	2700	92.5
Formerly in a union/living with a man	199	6.8
Number of children ever born		
One	705	24.2
Two	610	20.9
Three	436	14.9
Four or more	1167	40
Household wealth index		
Poorest	594	20.36
Poorer	503	17.24
Middle	457	15.66
Richer	460	15.76
Richest	904	30.98

*Catholic or traditional or wakefata **SNNPR: South Nation, Nationalities, and People Region.

### Obstetric-related characteristics of participants

Two-thirds, 1934 (66.3%) of the women had a history of births in the past five years and 2063 (70.7%) of the women had received ANC from a skilled provider. Among those who got ANC services, 1656 (56.8%) had four or more visits. One thousand two hundred and forty-five (43%) had their first ANC visit in the first three months of pregnancy. Of women who received ANC, 2635 (90.3%) had their blood pressure checked, 2412 (82.7%) had a blood sample taken, and 2292 (78.5%) had a urine sample taken “Table 2.”

**Table 2:** Obstetric-related characteristics of participants in Ethiopia, 2019E_DHS (n = 2918).

Variables	Frequency	Percent
Frequency of ANC visit		
1	141	4.8
2	353	12.1
3	768	26.3
4+	1656	56.8
Received ANC from a skilled provider		
Yes*	2063	70.7
No	855	29.3
Place of ANC follow-up		
Government health facility	2716	93.1
Private hospital	114	3.9
NGO health facility	88	3
Components of ANC service		
Blood pressure measured	2635	90.3
Blood sample taken	2412	82.7
Urine sample taken	2292	78.5
Given or bought iron tablets/ syrup	2214	75.9
Nutritional counseling	2088	71.6
Told about Signs of pregnancy complications or dangerous signs of pregnancy	1648	56.5
Births in the last five years		
1	1934	66.3
2	865	29.6
3	109	3.7
4	10	0.4
Currently pregnant women		
No or unsure	2595	88.9
Yes	323	11.1
Gestational Age at 1^st^ ANC visit (n = 323)		
≤3 months	66	20.4
4-6 months	121	37.5
7 months	44	13.6
8 + months	92	28.5

Hint ANC: Antenatal care; * Skilled providers include doctors, nurses, midwives, health officers, and health extension workers.

### Adequacy and consistency of antenatal care services in Ethiopia

Nationally provided antenatal care service was evaluated in terms of adequacy and consistency of ANC services based on the availability of information from mini-EDHS 2019. About 1656 (56.8%) women reported that they had at least four ANC visits from skilled attendants during their recent pregnancy, while only 1245 (43%) women-initiated ANC visits during the first trimester of gestation. Further, 1046 (35.8%) of the women received all six routine components of ANC services. Among those currently pregnant women, only 234(72.4%) received consistent antenatal care services “Table 3.”

**Table 3:** Consistency of ANC Services in Ethiopia (n = 2918).

Variables	Frequency	Percent
Initiation time ANC visit during the recent pregnancy		
Early	1245	42.7
Late	1673	57.3
Number of ANC visits		
< 4 visits	1262	43.2
>=4 visits	1656	56.8
Components of ANC Services		
Sufficient	1046	35.8
Insufficient	1872	64.2
Overall Consistency of ANC Services		
Consistent ANC services	234	72
Inconsistent ANC services	89	28

**Fig 1:**
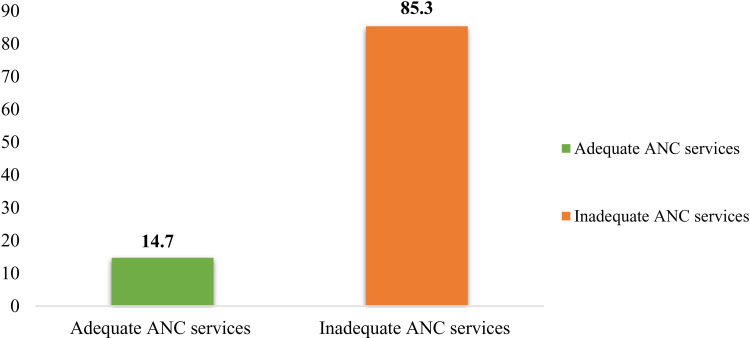
Overall adequacy of antenatal care services among pregnant women in Ethiopia.

More than one-fifth (14.7%) of women were received adequate antenatal care (ANC) in Ethiopia “Fig 1.”

### Regional disparities of adequacy of ANC services in Ethiopia

The ANC services’ adequacy was far less than 50%. It was higher in Addis Ababa, 88 (39.1%), and lowest in SNNPR, 20 (6.2%) and Gambela, 23 (8.6%) “Table 4.”

**Table 4:** Regional comparison of adequacy of ANC services in Ethiopia (n = 2918).

Variables		Regional comparison of ANC adequacy	
		Adequate (%)	Inadequate (%)
**Region**	Tigray	71(22.1)	251(77.9)
	Afar	39(17)	191(83)
	Amhara	64(19.2)	272(80.8)
	Oromia	38(11)	308(89)
	Somali	8(9.3)	78(90.7)
	Benishangul	34(11.4)	265(88.6)
	SNNPR	20(6.2)	306(93.8)
	Gambela	23(8.6)	245(91.4)
	Harari	79(32.1)	168(67.9)
	Addis Ababa	88(39.1	139(60.9)
	Dire Dawa	71(30.9)	161(69.1)

### Regional disparities of provision of consistent ANC services in Ethiopia

**Fig 2:**
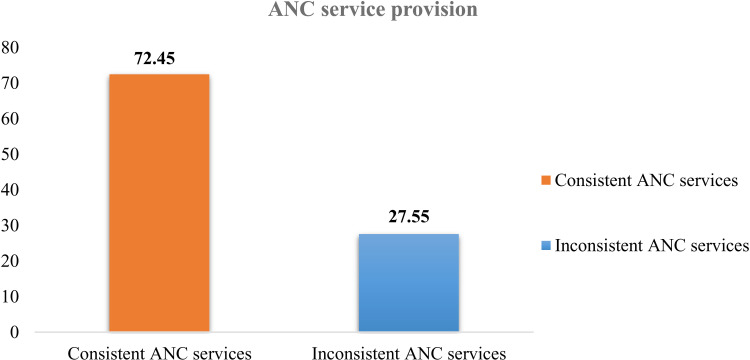
Consistency of prenatal care services among pregnant women in Ethiopia.

The study evaluated the overall consistency of antenatal care (ANC) services, with 233 individuals receiving consistent ANC services and 90 individuals receiving inconsistent ANC services. The data indicates that 72.45% of the women received consistent ANC services “Fig 2.”

The assessment of ANC service provision considered visit frequency, first initiation time, and completeness of services. Addis Ababa demonstrated the highest level of consistency in ANC service provision at 54.2%, followed by Dire Dawa at 45.5%. Benishangul and Harar had consistency rates of 34.55% and 34.3%, respectively. Conversely, Oromia (13.5%) and SNNPR (13.9%) exhibited poor ANC service consistency, ranking among the lowest in this aspect. It is evident that targeted efforts are needed to improve ANC service consistency in regions with lower rates “Table 5.”

**Table 5:** Provision of consistent ANC service Regional Comparison of women of reproductive age in Ethiopia, 2024.

Variables		Received consistent ANC service (n = 323)	
		No (%)	Yes (%)
**Region**	Tigray	27(75)	9(25)
	Afar	31(79.5)	8(20.5)
	Amhara	13(68.4)	6(31.6)
	Oromia	32(86.5)	5(13.5)
	Somali	10(76.9)	3(23.1)
	Benishangul	19(65.5)	10(34.5)
	SNNPR	31(86.1)	5(13.9)
	Gambela	19(82.6)	4(17.4)
	Harari	23(65.7)	12(34.3)
	Addis Ababa	11(45.8)	13(54.2)
	Dire Dawa	17(54.8)	15(45.5)
Overall consistency		233(72.45)	90(27.55)

### Factors associated with adequacy of ANC services

Logistic regression analyses were conducted to identify factors linked to the adequacy of ANC services, using a 95% confidence interval and a significance level of P < 0.05. The final model indicated that education level, region, wealth, and literacy were key factors affecting the adequacy of ANC services in Ethiopia. Women with higher education were almost twice as likely to receive adequate ANC services compared to those without formal schooling [AOR = 1.77, 95% CI: (1.02, 3.09)]. Regional disparities were also significant: women from the Oromia region had a 51% lower likelihood (AOR = 0.49, 95% CI: 0.31, 0.79), from the Benishangul region a 59% lower likelihood (AOR = 0.41, 95% CI: 0.24, 0.69), from the SNNPR region a 70% lower likelihood (AOR = 0.30, 95% CI: 0.17, 0.53), and from the Gambela region a 71% lower likelihood (AOR = 0.29, 95% CI: 0.15, 0.54) of receiving adequate ANC services compared to women from the Tigray region. The wealth index was another significant factor. Women in the poorer, middle, richer, and richest wealth categories had greater odds of receiving adequate ANC services, with adjusted odds ratios of [AOR = 1.96, 95% CI: (1.15, 3.34)], [AOR = 2.28, 95% CI: (1.35, 3.87)], [AOR = 2.52, 95% CI: (1.49, 4.24)], and [AOR = 3.66, 95% CI: (2.10, 6.37)], respectively, when compared to women in the poorest wealth category “Table 6.”

**Table 6:** Multiple logistic regression analysis of the adequacy of ANC services in Ethiopian, 2019 E_DHS (n = 2918).

Variables		Coef.	AOR (95% CI)	P-value
Educational status	No formal education		1	
	Primary education	0.02	1.02 (0.68, 1.52)	0.94
	Secondary education	0.23	1.26 (0.74, 2.12)	0.39
	Higher education	0.57	1.77 (1.02, 3.09)	0.04*
Age group (years)	15–24 years		1	
	25–34 years	0.06	1.07 (0.80, 1.42)	0.67
	35–49 years	0.09	1.10 (0.71, 1.70)	0.66
Household wealth index	Poorest		1	
	Poorer	0.67	1.96 (1.15, 3.34)	0.01*
	Middle	0.83	2.28 (1.35, 3.87)	0.01*
	Richer	0.92	2.52 (1.49, 4.24)	0.01*
	Richest	1.30	3.66 (2.10, 6.37)	0.01*
Residence	Urban		1	
	Rural	−0.18	0.83 (0.58, 1.20)	0.32
Region	Tigray		1	
	Afar	0.01	1.01 (0.59, 1.73)	0.98
	Amhara	−0.17	0.85 (0.55, 1.30)	0.45
	Oromia	−0.71	0.49 (0.31, 0.79)	0.01*
	Somali	−0.73	0.48 (0.18, 1.33)	0.16
	Benishangul	−0.90	0.41(0.24, 0.69)	0.01*
	SNNPR	−1.20	0.30 (0.17, 0.53)	0.01*
	Gambela	−1.25	0.29 (0.15, 0.54)	0.01*
	Harari	−0.20	0.82 (0.53, 1.28)	0.38
	Addis Adaba	0.02	1.02 (0.66, 1.59)	0.93
	Dire Dawa	−0.21	0.81 (0.52, 1.28)	0.37
Total children ever born**	β = −0.010		0.99 (0.92, 1.07)	0.79

Hint: 1= Reference group * = significant association at p-value <0.05 ** =Continuous variables.

### Factors associated with the consistency of ANC services in Ethiopia

Women with higher levels of education were more likely to receive consistent antenatal care (ANC) compared to those with no education. Women attended secondary education [AOR: 11.02 (95% CI: 1.47–26.67) and higher education [AOR: 15.73 (95% CI: 2.42–31.13) were 11 and 16 times more likely to received consistent ANC than women with no education respectively.

Women from Addis Ababa were 7.79 times more likely to receive consistent antenatal care (ANC) compared to women from Tigray [AOR = 2.79, 95% CI: (0.803, 9.658)]. Similarly, women from Dire Dawa were 2.67 times (AOR: 2.67 (95% CI: 0.85, 8.32) more likely to receive consistent ANC than women from Tigray “Table 7.”

**Table 7:** Factors associated with the Consistency of ANC services in Ethiopia, 2019.

Variables Categories		AOR	[95% CI]		P-value
	No education	1			
Educational Attainment	Primary education	1.27	0.54	2.99	0.58
	Secondary education	11.02	1.49	26.67	0.02*
	Higher education	15.73	2.42	31.13	0.01*
Region	Tigray	1			
	Afar	1.04	0.32	3.38	0.95
	Amhara	1.89	0.52	7.09	0.34
	Oromia	0.56	0.15	2.07	0.39
	Somali	1.02	0.19	5.24	0.99
	Benishangul	1.61	0.49	5.22	0.43
	SNNPR	0.49	0.13	1.86	0.30
	Gambela	0.40	0.08	2.07	0.27
	Harari	1.96	0.64	5.99	0.24
	Addis Adaba	2.79	0.80	9.66	0.04*
	Dire Dawa	2.67	0.85	8.32	0.03*

*Significant association at p-value <0.05.

## Discussion

The study utilized 2019 Mini-EDHS data to assess the quality of prenatal care services in Ethiopia. The results showed that only 14.7% of women received adequate ANC services, indicating significant deficiencies in the national ANC delivery system. Interestingly, there was a notable difference in ANC service adequacy between the national findings and a prior study conducted in Southern Ethiopia, which reported a lower rate of 6.2%. This disparity could be attributed to the differing scopes of the studies, with the national study encompassing a larger geographic area while the Southern Ethiopia study focused specifically on the SNNP region [14,15]. Apart from the results that were previously mentioned, the study also showed that a considerable percentage of Ethiopian women did not make full use of prenatal care facilities. In particular, the findings showed that 66.3% of pregnant women did not have ANC during the first trimester, a critical time for early identification and treatment. In addition, 22.3% of the pregnant women had insufficient antenatal care, receiving less than four visits during their pregnancy [16].

The study discloses vital statistics regarding the initiation of antenatal care [17] and the number of ANC visits among women. The findings indicate that only 42.7% of women in Ethiopia initiated ANC during the first trimester, which is considered early and optimal for detecting and addressing potential pregnancy-related issues. Additionally, 43.2% of women had at least four ANC visits throughout pregnancy. Comparing these results to similar data from Nigeria, it is noteworthy that Ethiopia shows higher rates of ANC initiation in the first trimester. In Nigeria, only 18% of women started ANC during the first trimester [18], suggesting that Ethiopia has made relatively better progress in promoting early ANC engagement.

There are regional disparities in the proportion of women receiving adequate antenatal care (ANC) services in Ethiopia. Tigray, Harari, and Addis Ababa have higher rates, while SNNPR, Oromia, and Somalia report lower rates. These differences are likely due to variations in resources, infrastructure, and access to ANC services. Research indicates that factors such as socioeconomic status, healthcare facility accessibility, geographic location, and transportation significantly influence women’s access to and use of ANC services [5,9,13,15,19]. Addressing these disparities requires targeted interventions to improve healthcare infrastructure, enhance the availability and accessibility of ANC services, and reduce socioeconomic barriers. Allocating resources and implementing strategies in regions with lower ANC adequacy rates will help improve maternal and child health outcomes across the country.

The study highlights the significance of educational status as a determinant of antenatal care service adequacy, with individuals having higher education demonstrating higher odds of receiving consistent ANC services. This underscores the importance of educational attainment in accessing and receiving adequate ANC. This result supports findings from Ethiopia [19], other sub-Saharan African research [20], a Study in Uganda [21], and a study in Nigeria [22]. The study also reveals a strong association between the household wealth index and ANC service adequacy, indicating that the odds of receiving consistent ANC services also increase as wealth increases. This result supports findings from [21,22]. This finding highlights the role of socioeconomic factors, particularly wealth, in accessing and utilizing ANC services effectively. Moreover, notable regional differences were observed, with individuals in certain regions, such as Oromia, Benishangul, SNNPR, and Gambela, having lower odds of receiving consistent ANC services than those in Tigray. These findings emphasize the need for targeted interventions and resource allocation to address regional disparities and ensure equitable access to ANC services across Ethiopia.

The finding of this study showed only 28% of women did not receive consistent prenatal care. Comparably, lower ANC coverage rates are problematic in several other Arabic nations. As an illustration, Morocco’s coverage rate was 60.9%, whereas Mauritania’s was 52.4%, and Saudi Arabia had a 79.9% ANC coverage rate in 2019 [17,23]. In contrast, some countries, such as Bahrain and the United Arab Emirates (UAE), stand out with exceptional ANC coverage, consistently nearing 100% [23]. The rates of women in Tigray and Afar who did not receive consistent ANC were 75% and 79.5%, respectively. Significant discrepancies were also seen in Oromia, Somali, SNNPR, and Gambela, where many women needed to receive ANC services consistently. Regional disparities in consistent ANC service provision result from differences in healthcare infrastructure, resource allocation, access to facilities, transportation challenges, socioeconomic conditions, and cultural practices. Targeted interventions and investment in healthcare infrastructure are crucial in regions with lower service provision rates. Improving accessibility, raising awareness about ANC attendance, and addressing socioeconomic barriers can enhance access to ANC services. Addressing these disparities will promote equitable access to high-quality ANC services, improving Ethiopia’s maternal and child health outcomes.

The study findings underscore the significance of educational attainment in the consistency of antenatal care services. Individuals with secondary education demonstrated significantly higher odds of receiving consistent ANC services than those without education, and individuals with higher education had even higher odds of ANC service consistency. This significant impact on educational level is similar to the study conducted in Saudi Arabia [17,23]. Regarding regional differences, individuals residing in Tigray had the highest likelihood of receiving consistent ANC services, while no statistically significant differences were observed in other regions compared to Tigray. These results emphasize the importance of educational attainment in ensuring ANC service consistency and suggest that regional disparities in Ethiopia have an insignificant impact on ANC service provision when compared to Tigray.

The implication of this study is that it discloses critical gaps in the adequacy and consistency of antenatal care services in Ethiopia, with noticeable disparities linked to region, education, literacy and wealth. Although many women are accessible to skilled ANC providers, few receive complete or adequate care services which indicate a quality shortfall in service delivery. Findings of this study indicated the need for focused interventions to advance Antenatal care (ANC) quality and it services, address regional and socioeconomic discrepancies and invest in women’s literacy and education. Moreover, a move from emphasizing ANC coverage alone to enhancing service quality and equity is crucial for improving maternal and child health outcomes in Ethiopia.

### Limitations of the study

The study relied on self-reported data, which may be subject to recall bias. The study also needed to explore why ANC service use varies by region. Researchers need to conduct further research to understand the causes behind the variations.

## Conclusions

In conclusion, the study highlights the inadequate and inconsistent ANC services in Ethiopia. Regional disparities in ANC provision and factors such as education, wealth, and geographic location contribute to disparities in service utilization. It is crucial to promote ANC utilization, particularly among less educated and economically disadvantaged women, with a particular focus on rural areas. Improving accessibility and quality of ANC services in rural areas should be prioritized to ensure equitable access to care. Targeted interventions are needed to address the service gaps experienced by women with lower education levels, including effective communication strategies to address misconceptions and concerns. Moreover, addressing the significant regional differences in ANC consistency and adequacy requires implementing focused measures to bridge this gap. By improving ANC services, maternal and child health outcomes can be significantly enhanced throughout Ethiopia.

## References

[1] WHO. WHO recommendations on antenatal care for a positive pregnancy experience. 16.12. 2016. 28079998

[2] Abdo R, Demelash M, Seid AM, Mussema A. First trimester antenatal care contact in Africa: a systematic review and meta-analysis of prevalence and contributing factors. BMC Pregnancy Childbirth. 2023;23(1):742. doi: 10.1186/s12884-023-06034-1 37858033 PMC10585910

[3] WHO. Trends in maternal mortality 2000 to 2020: estimates by WHO, UNICEF, UNFPA, World Bank Group and UNDESA/Population Division. Executive summary. Geneva: World Health Organization; 2023.

[4] Tesfaye G, Chojenta C, Smith R, Loxton D. Application of the Andersen-Newman model of health care utilization to understand antenatal care use in Kersa District, Eastern Ethiopia. PLoS One. 2018;13(12):e0208729. doi: 10.1371/journal.pone.0208729 30521640 PMC6283597

[5] Areru HA, Dangisso MH, Lindtjørn B. Large local variations in the use of health services in rural southern Ethiopia: an ecological study. PLOS Glob Public Health. 2022;2(5):e0000087. doi: 10.1371/journal.pgph.0000087 36962269 PMC10021478

[6] Pervin J, Venkateswaran M, Nu UT, Rahman M, O’Donnell BF, Friberg IK, et al. Determinants of utilization of antenatal and delivery care at the community level in rural Bangladesh. PLoS One. 2021;16(9):e0257782. doi: 10.1371/journal.pone.0257782 34582490 PMC8478219

[7] Tadele F, Getachew N, Fentie K, Amdisa D. Late initiation of antenatal care and associated factors among pregnant women in Jimma Zone Public Hospitals, Southwest Ethiopia, 2020. BMC Health Serv Res. 2022;22(1):632. doi: 10.1186/s12913-022-08055-6 35549700 PMC9097060

[8] Ethiopian Public Health Institute (EPHI), ICF. Ethiopia Mini demographic and health survey 2019: final report. Rockville, Maryland, USA: EPHI and ICF; 2021.

[9] Negero MG, Sibbritt D, Dawson A. Women’s utilisation of quality antenatal care, intrapartum care and postnatal care services in Ethiopia: a population-based study using the demographic and health survey data. BMC Public Health. 2023;23(1):1174. doi: 10.1186/s12889-023-15938-8 37337146 PMC10278283

[10] Ataguba JE-O. A reassessment of global antenatal care coverage for improving maternal health using Sub-Saharan Africa as a case study. PLoS One. 2018;13(10):e0204822. doi: 10.1371/journal.pone.0204822 30289886 PMC6173396

[11] Ekholuenetale M, Nzoputam CI, Barrow A, Onikan A. Women’s enlightenment and early antenatal care initiation are determining factors for the use of eight or more antenatal visits in Benin: further analysis of the Demographic and Health Survey. J Egypt Public Health Assoc. 2020;95(1):13. doi: 10.1186/s42506-020-00041-2 32813174 PMC7364685

[12] WHO. Family planning a global handbook for providers: evidence-based guidance developed through worldwide collaboration. 2022. https://fphandbook.org/sites/default/files/WHO-JHU-FPHandbook-2022Ed-v221114b.pdf

[13] Tadele A, Teka B. Adequacy of prenatal care services and associated factors in Southern Ethiopia. Arch Public Health. 2021;79(1):94. doi: 10.1186/s13690-021-00614-3 34099020 PMC8183068

[14] Kare AP, Gujo AB, Yote NY. Quality of antenatal care and associated factors among pregnant women attending government hospitals in Sidama Region, Southern Ethiopia. SAGE Open Med. 2021;9:20503121211058055. doi: 10.1177/20503121211058055 34868590 PMC8640313

[15] Wuneh AD, Bezabih AM, Okwaraji YB, Persson LÅ, Medhanyie AA. Wealth and education inequities in maternal and child health services utilization in rural Ethiopia. Int J Environ Res Public Health. 2022;19(9):5421. doi: 10.3390/ijerph19095421 35564817 PMC9099508

[16] Yaya S, Bishwajit G, Ekholuenetale M, Shah V, Kadio B, Udenigwe O. Timing and adequate attendance of antenatal care visits among women in Ethiopia. PLoS One. 2017;12(9):e0184934. doi: 10.1371/journal.pone.0184934 28922383 PMC5602662

[17] Alanazy W, Rance J, Brown A. Exploring maternal and health professional beliefs about the factors that affect whether women in Saudi Arabia attend antenatal care clinic appointments. Midwifery. 2019;76:36–44. doi: 10.1016/j.midw.2019.05.012 31154158

[18] Nigeria Demographic and Health Survey. National Population Commission Abuja, Nigeria The DHS Program ICF. 174-175. 2018.

[19] Belay AT, Fenta SM, Birhan Biresaw H, Abebaw Moyehodie Y, Melkam Yelam M, Mekie M. The magnitude of optimal antenatal care utilization and its associated factors among pregnant women in South Gondar Zone, Northwest Ethiopia: a cross-sectional study. Int J Reprod Med. 2022;2022:1415247. doi: 10.1155/2022/1415247 36092776 PMC9463004

[20] Bobo FT, Asante A, Woldie M, Hayen A. Poor coverage and quality for poor women: inequalities in quality antenatal care in nine East African countries. Health Policy Plan. 2021;36(5):662–72. doi: 10.1093/heapol/czaa192 33822943

[21] Towongo MF, Ngome E, Navaneetham K, Letamo G. A secondary analysis of the factors associated with women´s adequate utilization of antenatal care services during their last pregnancy in Uganda. BMC Pregnancy Childbirth. 2023;23(1):692. doi: 10.1186/s12884-023-05994-8 37749492 PMC10521507

[22] Agho KE, Ezeh OK, Ogbo FA, Enoma AI, Raynes-Greenow C. Factors associated with inadequate receipt of components and use of antenatal care services in Nigeria: a population-based study. Int Health. 2018;10(3):172–81. doi: 10.1093/inthealth/ihy011 29562242

[23] Alqifari SF. Antenatal care practices: a population-based multicenter study from Saudi Arabia. Int J Womens Health. 2024;16:331–43. doi: 10.2147/IJWH.S452934 38444593 PMC10913596

