# Supplementary material for: Sufficiency and consistency of antenatal care services in Ethiopia: Evidence from Mini Ethiopian demographic and health survey
Source: PLoS One. 2025 Jun 6;20(6):e0319291. doi: 10.1371/journal.pone.0319291 (PMC12143500; doi:10.1371/journal.pone.0319291)
Supplement: Attachment — Submitted filename: Response to the reviewers comment_P1_2025.docx [file pone.0319291.s002.docx]

Dear reviewer, we would like to express our sincere gratitude for your constructive comments and appreciation. We have carefully addressed each of your comment point by point.

We have also highlighted all the changes we made to the main manuscript.

1. Consistency vs adequacy

Response: Methods_Line 142- Dear reviewer we have operationalized these two variables. They are used to measure to consistency of ANC services and adequacy of ANC services. They are used to indicate different outcome variables.

1. *Consistency of ANC visit: measured if the pregnant mother received services during antenatal care visits such as, identifying pre-existing health conditions, early detection of complications during pregnancy, health promotion and disease prevention, birth preparedness, and complication planning*
2. *Adequacy of antenatal care: The adequacy of antenatal care is measured using three indicators: early initiation of antenatal care (the first antenatal care visit made during the first trimester or three months), attending enough visits (at least four and above visits), and getting services at least once during the pregnancy care)*
2. Why guest were considered eligible for survey?

- Response: Methods_Line 123: We have addressed this comment, we paraphrased it.

*‘‘The data were gathered through interviews with women aged 15 to 49 years, who were long-term residents of the selected households.’’*

3. Please keep Study population consistent: is it last 1 year or 5 year

- Response: Methods_Line 108: Thank you, we have addressed it.
  *The study population consisted of women who had at least one antenatal care visit during their most recent birth within the five years preceding the 2019 Ethiopian Mini-Demographic and Health Survey.*

4. Tables: Please format table and keep decimal up to two places

- *Thank you, we have addressed it, we have corrected to two.*

5. Table 1: Please define household wealth index

- *Response: Methods_Line 153: Now we have added the operational definition of Wealth Index:*

*The wealth index weights were derived using principal component analysis (PCA) based on data collected from household assets, utilities, and other variables from the Ethiopian Demographic and Health Survey. PCA was used to create latent factors that represent wealth, with the first factor serving as the household wealth score. These scores were then divided into five quintiles: lowest, second, middle, fourth, and highest.*

6. Table 3: What is sufficient ANC services

Now we have added the operational definition of sufficient ante-natal care.

- *Methods_Line 146: Sufficient antenatal care: Sufficient antenatal care involves the recommended minimum of four timely visits, providing comprehensive services such as screenings, monitoring, and health counseling to ensure the well-being of both mother and baby.*

7. Regional Disparities of Provision of consistent ANC services in Ethiopia: Please mention denominator and %

*Response: Dear Reviewer, we have mentioned it in the table. A total of 323 individuals are included into the analysis. The number of individuals who are consistent and not consistent are mentioned in row (Table 5)*

8. Factors associated with Adequacy of ANC services: Is literacy level different from education level?

*Response: In this study educational status mean level of the education which the respondent attended.*

9. Discussion: what is acceptable ANC?

Discussion Line_292: Sorry we have corrected it to adequate.

*The study utilized 2019 Mini-EDHS data to assess the quality of prenatal care services in Ethiopia. The results showed that only 14.7% of women received adequate ANC services, indicating significant deficiencies in the national ANC delivery system.*

10. Rationale for regional disparities: Authors have mentioned, Socioeconomic status, healthcare facility accessibility, geographic location, and transportation can affect women's access to and utilization of ANC services. But some of these variables were not included in analysis
    Discussion line 313: Now, we have corrected it, here we want to show findings from other studies.

*……Research indicates that factors such as socioeconomic status, healthcare facility accessibility, geographic location, and transportation significantly influence women's access to and use of ANC services….*

11. Limitation: The study did not explore the reasons behind the regional disparities in ANC adequacy.

*Response: Dear Reviewer, this study uses DHS data, a national survey conducted every five years. Some variables are selected at the national level to guide the survey. We, the authors, have no authority to add variables to further explore the reasons behind the regional disparities in ANC adequacy, as the data is collected by central statistics. We have also acknowledged this as a limitation in our report.*

*Line 364: Limitations of the study*

*The study relied on self-reported data, which may be subject to recall bias. The study also needed to explore why ANC service use varies by region. Researchers need to conduct further research to understand the causes behind the variations.*
